# Supplementary material for: Novel Platform for Regulation of Extracellular Vesicles and Metabolites Secretion from Cells Using a Multi-Linkable Horizontal Co-Culture Plate
Source: Micromachines (Basel). 2021 Nov 21;12(11):1431. doi: 10.3390/mi12111431 (PMC8623696; doi:10.3390/mi12111431)
Supplement: Supplementary file 1 [file micromachines-12-01431-s001.zip › micromachines-1405077-supplementary.pdf]

micromachines

Supplementary Information: Novel Platform for Regulation of Extracellular Vesicles and Metabolites Secretion from Cells Using a Multi-Linkable Horizontal Co-Culture Plate

Takeo Shimasaki [1,2,3,] *, Satoko Yamamoto [1], Risa Omura [1], Kagenori Ito [4,5], Yumiko Nishide [6], Hideki Yamada [6], Kazumi Ohtomo [6], Tomo Ishisaka [7], Keiichiro Okano [7], Takenori Ogawa [8], Hiroyuki Tsuji [7], Yoichi Matsuo [9], Toshinari Minamoto [2], Naohisa Tomosugi [1], Etienne Ferain [10] and Takahiro Ochiya [5,11]

[1] Medical Research Institute, Kanazawa Medical University, Uchinada 920-0293, Japan; s-yama@kanazawa-med.ac.jp (S.Y.); r-omura@kanazawa-med.ac.jp (R.O.); tomosugi@kanazawa-med.ac.jp (N.T.)
[2] Division of Translational and Clinical Oncology, Cancer Research Institute, Kanazawa University, Kanazawa 920-0934, Japan; minamoto@staff.kanazawa-u.ac.jp
[3] Ginrei Lab Inc., Uchinada 920-0293, Japan; info@ginreilab.com
[4] Department of Urology, Jikei University School of Medicine, Tokyo 105-0003, Japan
[5] Division of Cellular Signaling, National Cancer Center Research Institute, Tokyo 104-0045, Japan; ito.kagenori@gmail.com (K.I.)
[6] Shinko Chemical Co. Ltd., Kanazawa 920-0346, Japan; master@shinko-ccl.jp (Y.N.); hyamada@shinko-ccl.jp (H.Y.); ohtomo@shinko-ccl.jp (K.O.)
[7] Department of Head and Neck Surgery, Kanazawa Medical University, Uchinada 920-0293, Japan; tomo-n@kanazawa-med.ac.jp (T.I.); okano-k@kanazawa-med.ac.jp (K.O.); tsujih-i@kanazawa-med.ac.jp (H.T.)
[8] Department of Otolaryngology-Head and Neck Surgery, Gifu University Graduate School of Medicine, Gifu 501-1194, Japan; ogawa@gifu-u.ac.jp
[9] Department of Gastroenterological Surgery, Nagoya City University Graduate School of Medical Sciences, Nagoya 467-8601, Japan; matsuo@med.nagoya-cu.ac.jp
[10] it4ip S.A., Louvain-la-Neuve 1348, Belgium; ferain@it4ip.be
[11] Department of Molecular and Cellular Medicine, Institute of Medical Science, Tokyo Medical University, Tokyo 160-0023, Japan; tochiya@tokyo-med.ac.jp (T.O.)

* Correspondence: takeo@kanazawa-med.ac.jp; Tel.: +81-762-218-8312

Supplementary information

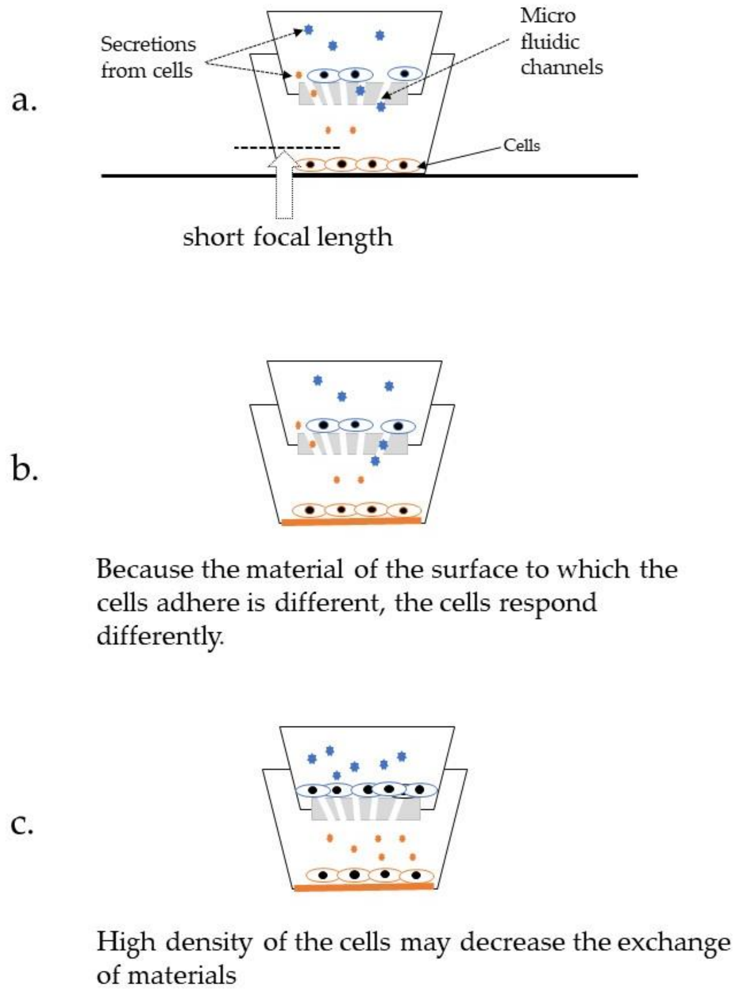

Figure S1. The three main problems associated with a vertical-type co-culture plate (VTCP). (a) The upper container cannot be observed because of the short focal length of the microscope. (b) The surface material to which the cells adhere differs, meaning that the cells may respond differently. (c) The high density of cells may prevent the exchange of materials.

.

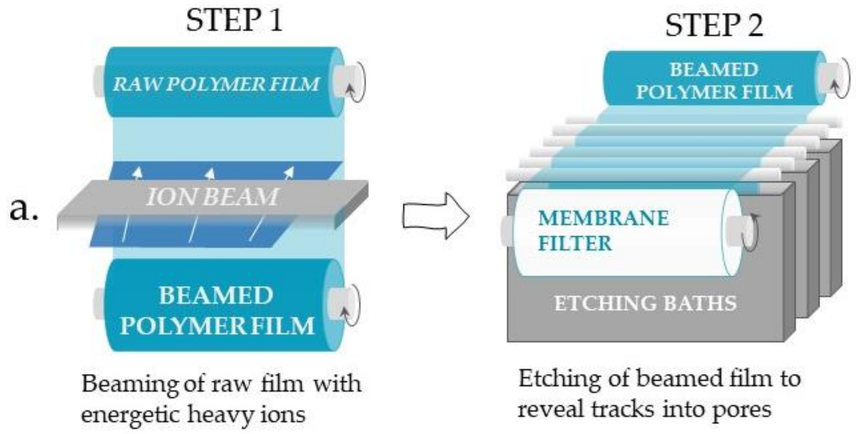

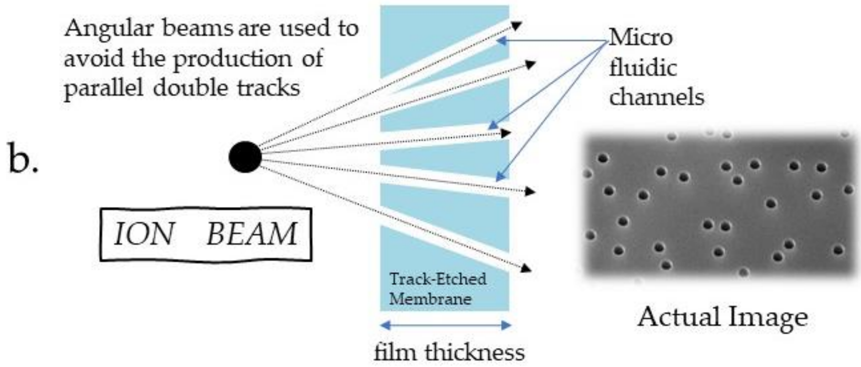

Figure S2. (a) Track-etched membranes were fabricated by irradiating a raw polymer film with energetic heavy ions. (b) The beamed film was then chemically etched to make uniform and precise pores. Pore density and pore orientation were determined during the beaming step, while the etching conditions defined the pore size.

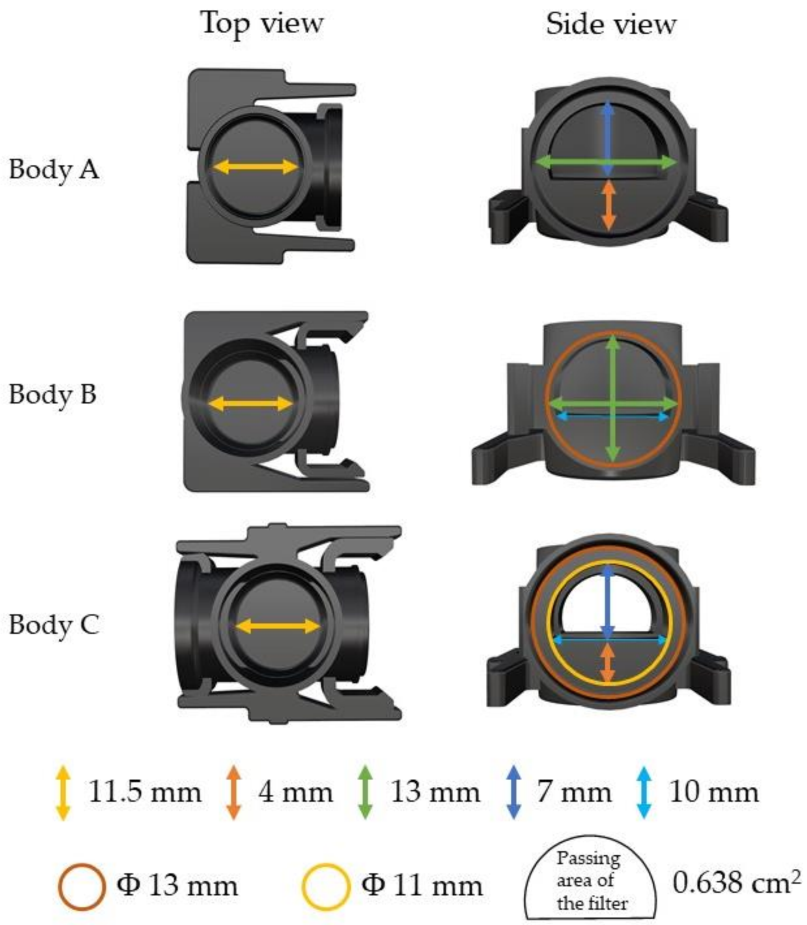

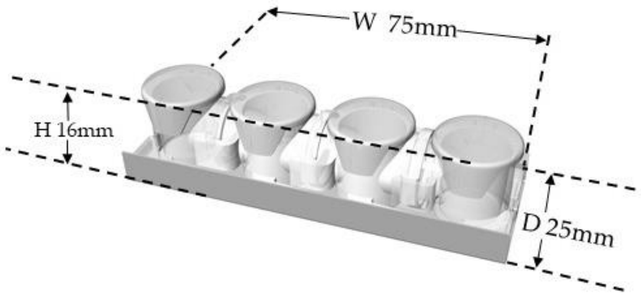

Figure S3. Size of each component. The size of each component is described in the figure. The top cover of the main body was made of polystyrene, the common sidewall cover was constructed using low-density polyethylene, and the O-ring was made of silicone. The O-ring was hydrophilized via corona discharge. All materials used for building the main body of the HTCP were sterilized with electron beams.

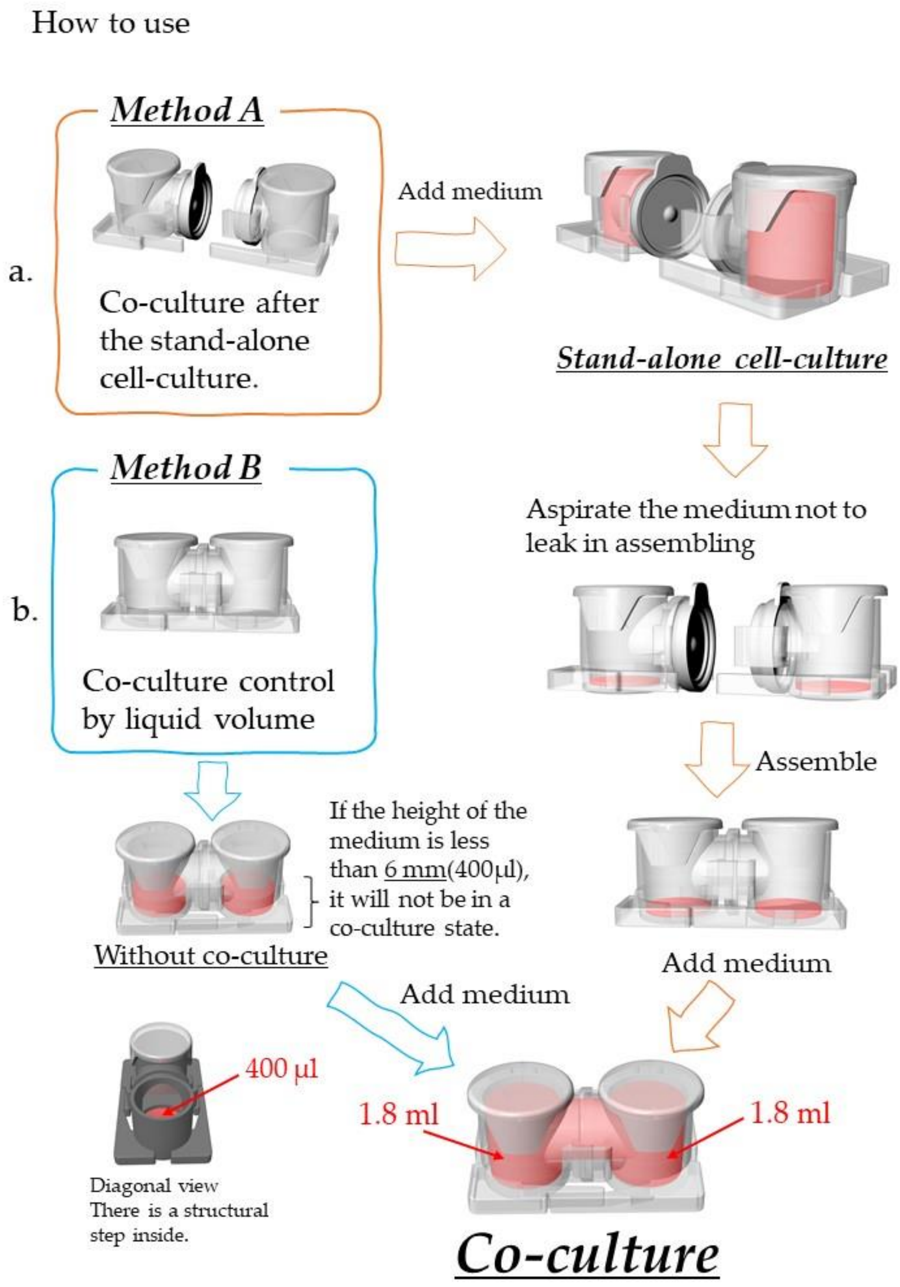

Figure S4. How to use. Methods A (Initial monoculture mode) and B (Con-trolling co-culture with medium volume). (a) In a stand-alone way, the cell types are cultured separately and then co-cultured by connecting the two containers (combined state) without dam-aging the cells. Bodies A and B are designed to allow the use of separated cell culturing. (b) In the connected mode, the two containers are joined from the beginning of cell culture. The height (volume) of the culture medium is used to control the amount of liquid that traverses the two containers, controlling the extent of co-culturing. Re-separation is possible. After reducing the amount of culture medium, one can separate bodies A and B by applying force to pull them apart to the left and right, and then cover the sides with a side cover and increase the amount of culture medium to create a monoculture. However, as this is a plastic product, recombination after separation could be problematic.

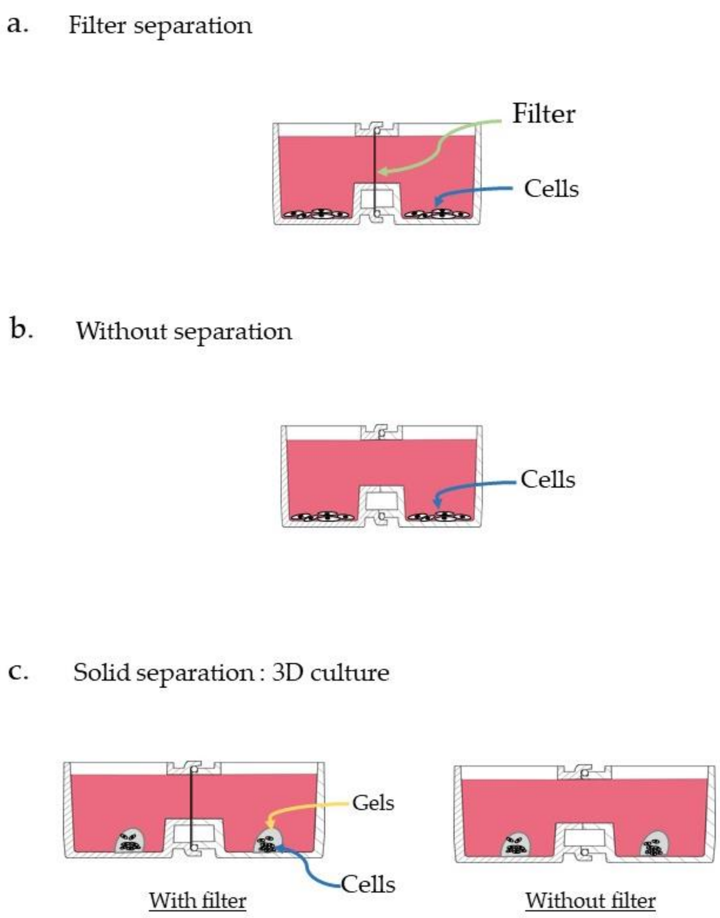

Figure S5. Schematic of various use methods: (a) filter separation, (b) without separation, and (c) solid separation with gels (three-dimensional culture).

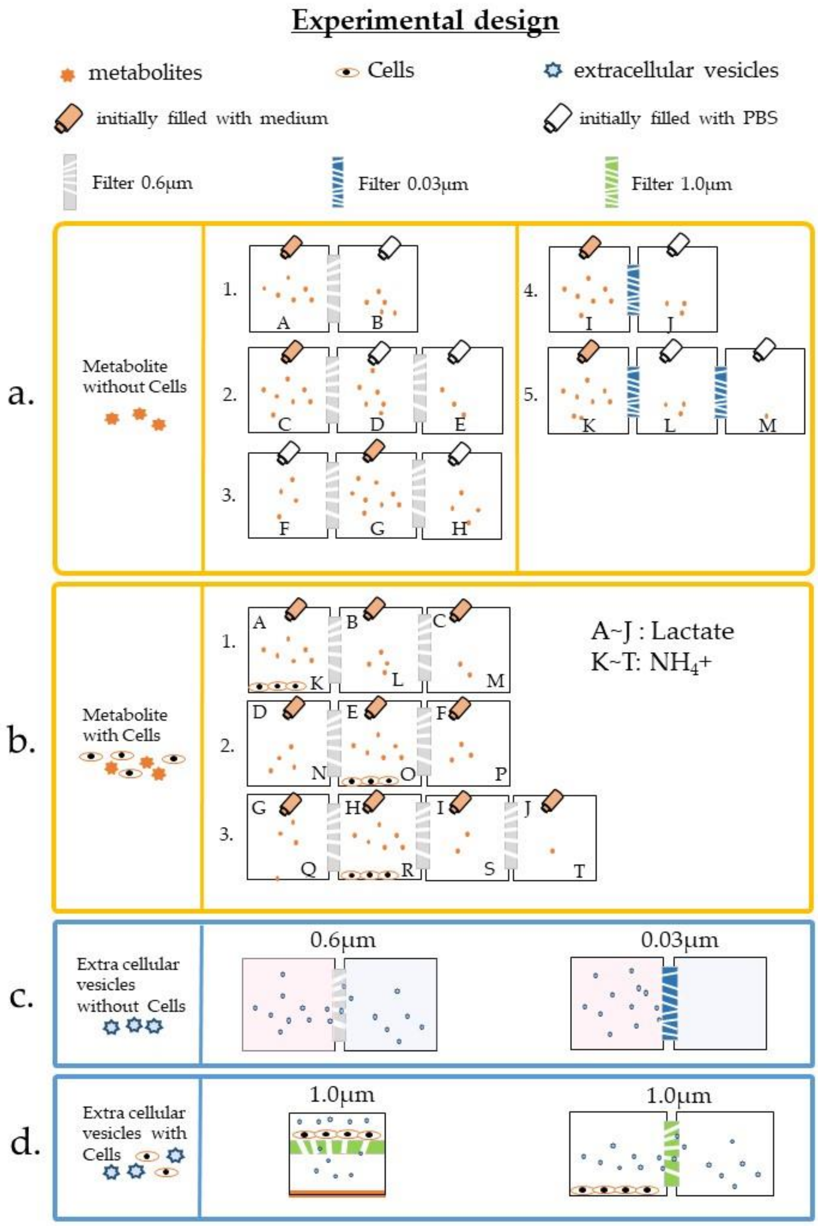

Figure S6. Summary of experimental design. (a) Differences in mass transfer due to the number of containers, position of the medium, and pore size of the filter were measured. All the measurements were performed under cell-free conditions. (b) Differences in metabolite concentrations due to differences in the positions of the containers in which the cells were placed were measured. (c) The filter passability of exosomes was measured. (d) Comparison of the passability of exosomes between VTCP and HTCP.

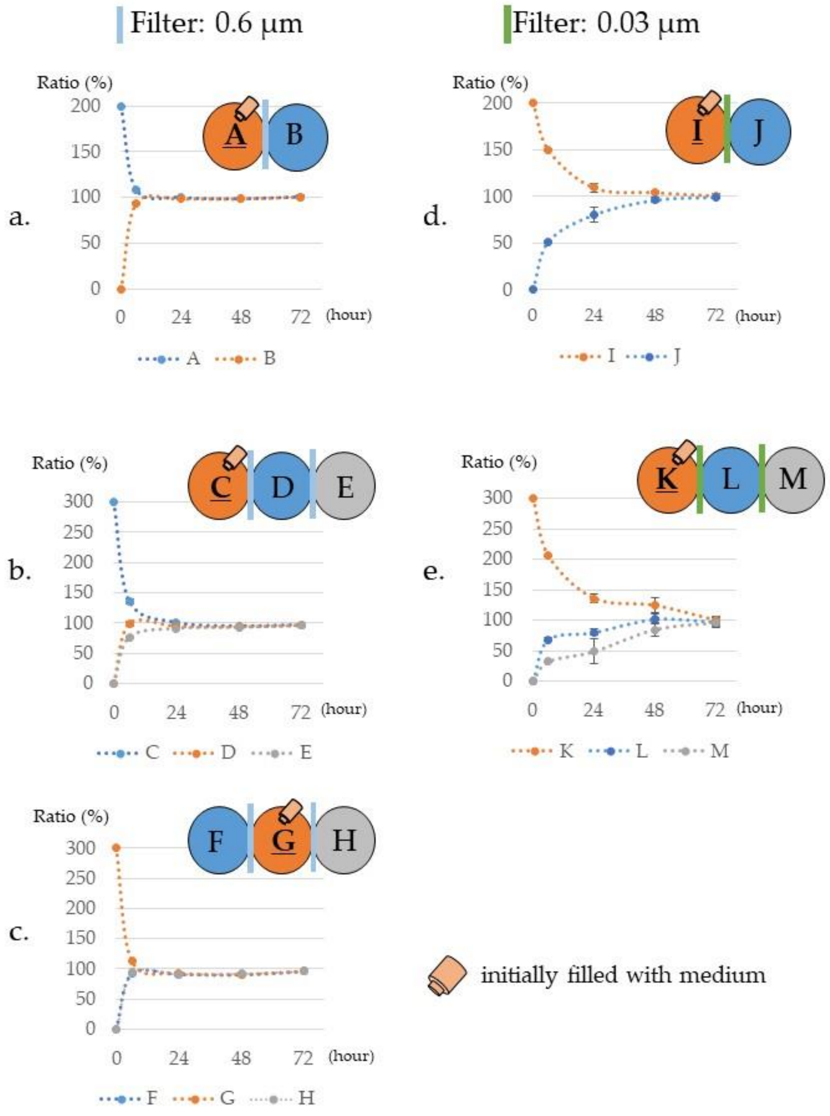

Figure S7. The results of NH4+ transfer without cells. Orange-colored wells with clip art of bottle were filled with medium including glucose. The remaining wells were filled with phosphate-buffered saline (PBS), which did not contain glucose. When all wells were mixed, the final concentration was converted to 100 %. The vertical axis shows the converted concentra-tion in %. Glucose concentration in the medium was measured after 6–72 h. The vertical axis is the concentration (%), and the horizontal axis is the time (h). Filter pore size is 0.6 μm or 0.03μm.

a.

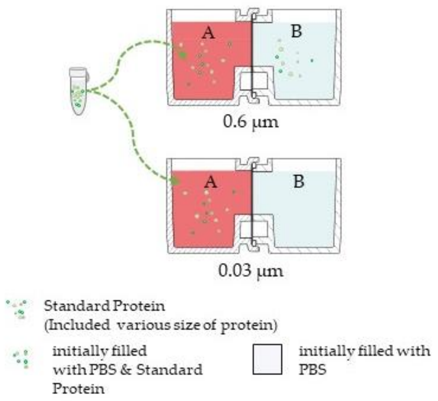

b.

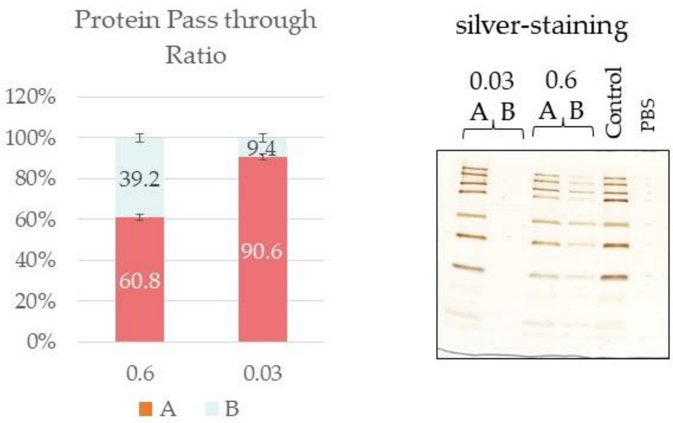

Figure S8. Migration of proteins through filters (A) Equal amounts of stand-ard proteins were added to one side of the horizontal type co-culture system (HTCP) to test the ability of proteins to pass through filters of different sizes. (B) Proteins passing through the 0.6 µm and 0.03 µm filters were measured with silver staining.

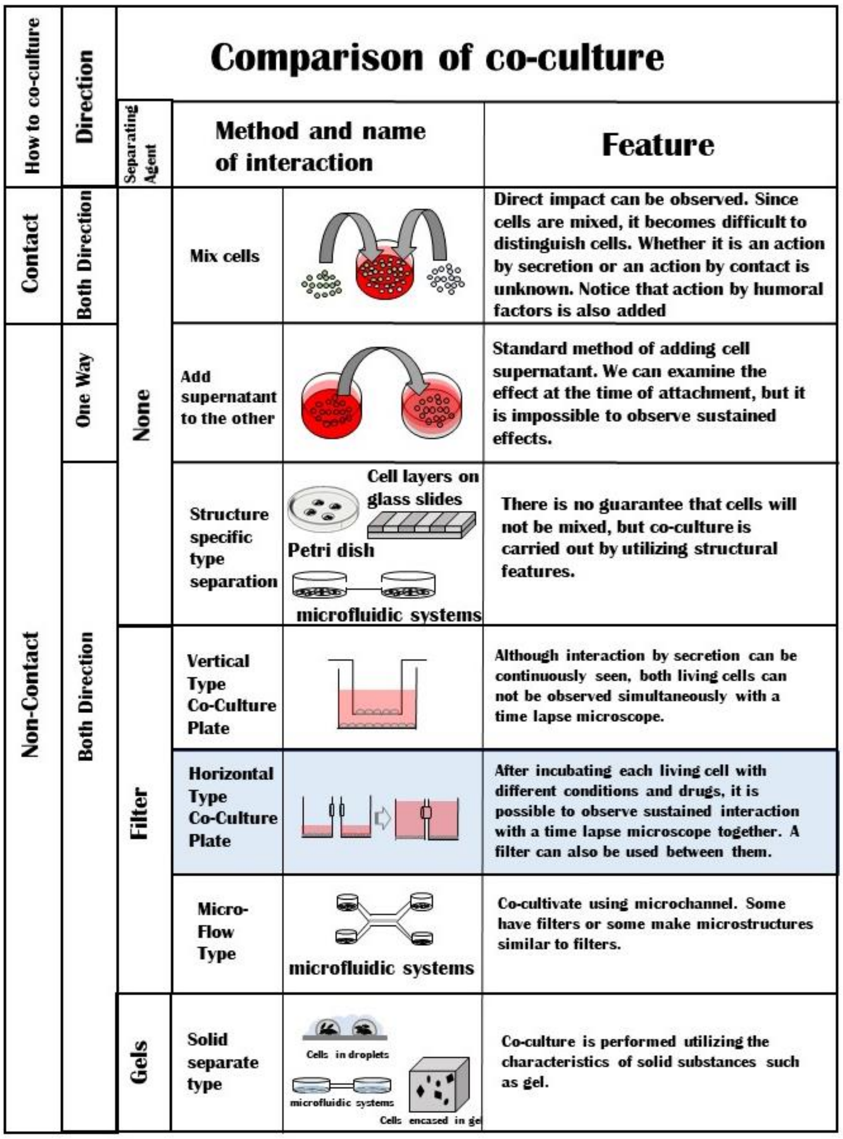

Figure S9. Various co-culture methods have been introduced in this study. Co-culture methods can be divided into two categories based on whether the cells are in direct contact or not. Next, they are divided by based on the nature of the material exchange i. e. bidi-rectional or unidirectional. Finally, different types of separation materials exist. Some use filters, while others use microfluidic channels, and some use no separating material. Modified from Shimasaki et al. [1] with permission.

Differences in Co-culture Vessels by Vertical and Horizontal Binding Directions.

Type of vessels<br>Connection type	VTCP<br>Vertical connection	HTCP<br>Horizontal connection
Image	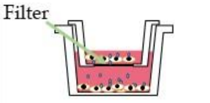	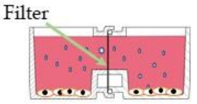
The morphology of the cells in the two chambers can be easily visualized by microscope	×<br>morphology of the cells in upper chamber can not be visualized.	○<br>Time-lapse video can be taken of the growing cell cultures in both chamber.
Both cell cultures grow on same type of plastic surface.	×	○
A variety of different filter pore sizes can be used; changing the maximum size of molecules shared between the two culture vessels.	△	○
Growth of cells do not prevent the exchange of liquid materials such as EVs by blocking the pore of filter	△	○
Multi connection	×	○

Figure S10. List of differences in features between VTCP and HTCP modified from Shimasaki et al. [1] with permission.

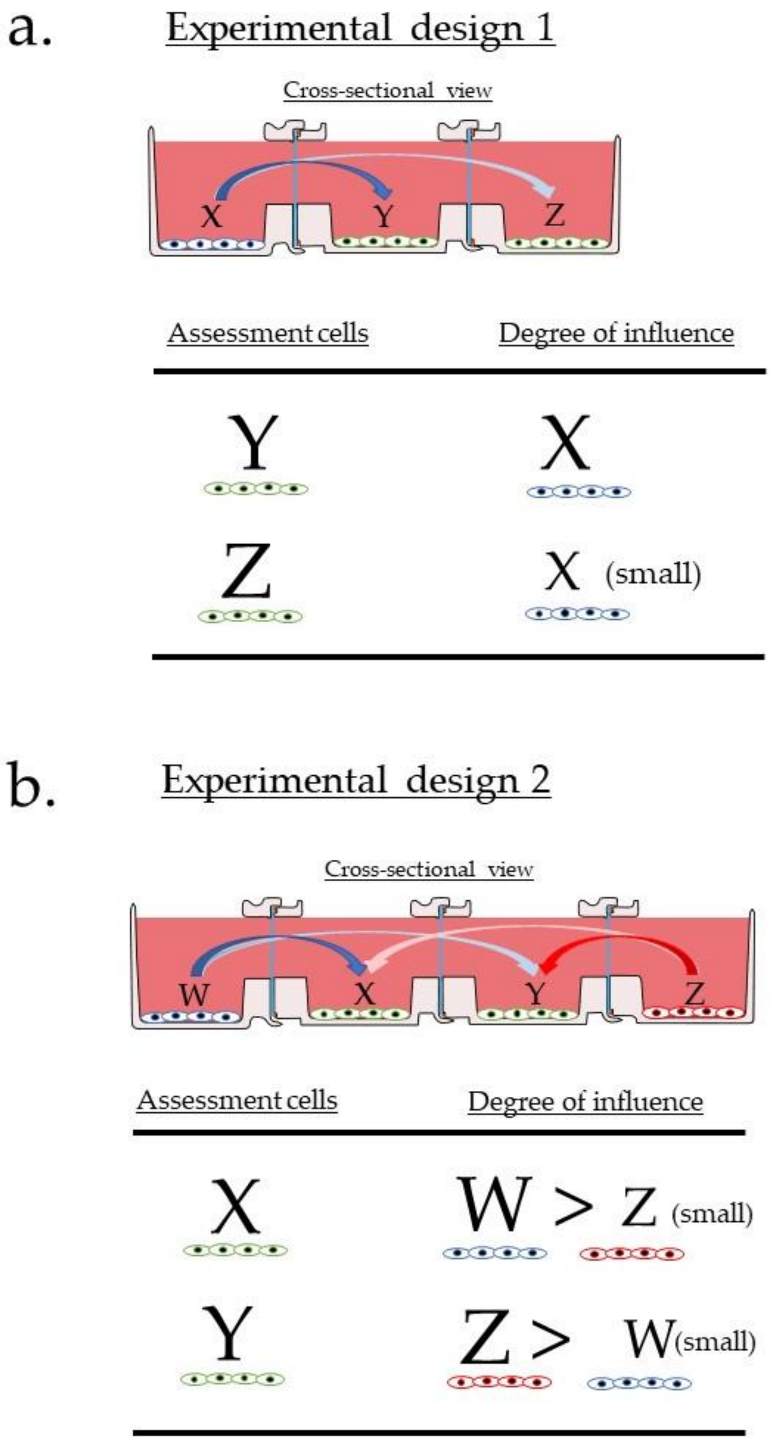

Figure S11. (a) Experimental design 1: A method to check for changes in the cells due to different degrees of influence. Different distances from the cells produce different concentrations, and changes due to different degrees of influence can be observed. (b) Experi-mental design 2: How to check the change in the two types of cells due to different degrees of in-fluence. For example, suppose X and Y are the same cells, and W and Z are different cells from X and Y. We can confirm that X and Y show different changes owing to the different influences of W and Z.

Filter material/ pore size/ density/ area

Material	Filter Size(Φ) (area)	Pore Size(Φ)	Pore density (Number)/ cm^2	Number of effective pores	Total area of effective pore area	Ratio of open pore area to filter area
Polycarbonate	13mm (1.327 cm^2)	0.03μm	$6.00x10^8$	$4.00x10^8$	$2.71x10^{-3}$ cm^2	0.424%
Polycarbonate	13mm (1.327 cm^2)	0.6μm	$4.00x10^8$	$2.55x10^8$	$7.22x10^{-1}$ cm^2	11.311%

Table S1. The density and number of pores in each filter were described. The area of the filter pores as a percentage of the effective filter area in the passage was calculated and expressed as the percentage of open pores.

(A) $y = 6E\text{-}05X^2 + 0.0027X + 0.0043$	$R^2 = 0.9999$
(B) $y = 5E\text{-}05X^2 + 0.0004X - 0.0014$	$R^2 = 0.9999$
(C) $y = 4E\text{-}05X^2 + 0.0001X - 0.0038$	$R^2 = 0.9987$
(D) $y = 4E\text{-}05X^2 + 0.0002X + 0.0062$	$R^2 = 0.9992$
(E) $y = 5E\text{-}05X^2 + 0.0005X + 0.0154$	$R^2 = 1$
(F) $y = 4E\text{-}05X^2 + 0.0004X + 0.0065$	$R^2 = 1$
(G) $y = -2E\text{-}06X^3 + 0.0003X^2 - 0.0065X + 0.0368$	$R^2 = 1$
(H) $y = -1E\text{-}06X^3 + 0.0002X^2 - 0.0046X + 0.04$	$R^2 = 1$
(I) $y = -2E\text{-}06X^3 + 0.0002X^2 - 0.0056X + 0.0325$	$R^2 = 1$
(J) $y = -2E\text{-}06X^3 + 0.0002X^2 - 0.007X + 0.0333$	$R^2 = 1$
(K) $y = -2E\text{-}06X^3 + 0.0002X^2 + 0.0003X + 0.139$	$R^2 = 1$
(L) $y = -3E\text{-}06X^3 + 0.0003X^2 - 0.004X + 0.1603$	$R^2 = 1$
(M) $y = -1E\text{-}06X^3 + 0.0001X^2 - 0.0002X + 0.1397$	$R^2 = 1$
(N) $y = -2E\text{-}06X^3 + 0.0002X^2 - 0.0029X + 0.1461$	$R^2 = 1$
(O) $y = -9E\text{-}07X^3 + 0.0001X^2 + 0.0013X + 0.1251$	$R^2 = 1$
(P) $y = -2E\text{-}06X^3 + 0.0002X^2 - 0.0029X + 0.149$	$R^2 = 1$
(Q) $y = -2E\text{-}06X^3 + 0.0003X^2 - 0.0045X + 0.1574$	$R^2 = 1$
(R) $y = -2E\text{-}06X^3 + 0.0003X^2 - 0.0041X + 0.1582$	$R^2 = 1$
(S) $y = -3E\text{-}06X^3 + 0.0003X^2 - 0.0053X + 0.1476$	$R^2 = 1$
(T) $y= -2E\text{-}06X^3 + 0.0003X^2 - 0.0046X + 0.1455$	$R^2 = 1$

Y(g/L) X (Hour)

Table S2. The list of correlation approximation equations and correlation coef-ficients is shown in Figure 6.

Condition	Figure No.	Objects	Location	Time									
				0 h	6 h	24 h	48 h	72 h		6 h	24 h	48 h	72 h
				mean	mean	mean	mean	mean		S.E.	S.E.	S.E.	S.E.
		Glucose 0.6μm	A	200	119.54766	99.363057	97.160883	97.121212		1.6	1.0	0.3	0.5
		Glucose 0.6μm	B	0	84.006462	94.267516	95.741325	96.969697		1.3990717	0.7297095	0.2731941	0.6943297
		Glucose 0.6μm	C	300	169.67419	110	96.897375	93.693694		3.7089345	1.6898057	1.8018698	1.4065311
		Glucose 0.6μm	D	0	93.233083	93.658537	93.556086	91.441441		4.186289	2.195122	1.6535091	0.7802031
		Glucose 0.6μm	E	0	46.115288	82.682927	90.930788	89.864865		0.4340979	1.2673542	0.7159905	0.6756757
		Glucose 0.6μm	F	0	84.210526	96.097561	97.852029	95.720721		0	1.1177014	1.0936935	1.4065311
Medium	Figure 4.	Glucose 0.6μm	G	300	139.84962	101.70732	98.806683	95.720721		1.5037594	1.2673542	1.4319809	1.7004132
		Glucose 0.6μm	H	0	83.9599	96.341463	98.329356	95.495495		0.4340979	1.5231702	1.8018698	1.0321116
				0 h	6 h	24 h	48 h	72 h		6 h	24 h	48 h	72 h
				mean	mean	mean	mean	mean		S.E.	S.E.	S.E.	S.E.
		Glucose 0.03μm	I	200	192.73021	166.40127	129.49527	115.60606		1.008885	1.9888529	2.8522305	1.1439143
		Glucose 0.03μm	J	0	11.470113	37.738854	65.457413	82.121212		0.2798143	0.8274128	4.8564051	3.4116152
		Glucose 0.03μm	K	300	294.48622	247.07317	189.02148	156.53153		2.4169551	1.5231702	6.9047619	6.8016526
		Glucose 0.03μm	L	0	18.045113	55.853659	80.668258	89.414414		2.2556391	10.094733	5.8314042	3.333029
		Glucose 0.03μm	M	0	0	4.3902439	27.684964	43.918919		0	2.6382083	5.3739047	6.4455351
					6 h	24 h	48 h	72 h		6 h	24 h	48 h	72 h
					mean	mean	mean	mean		S.E.	S.E.	S.E.	S.E.
		Lactate 0.6μm	A		0.0233333	0.1	0.2666667	0.4933333		0.008165	0.0141421	0.0285774	0.0432049
		Lactate 0.6μm	B		0.0033333	0.0366667	0.14	0.3		0.0040825	0.008165	0.0254951	0.0374166
		Lactate 0.6μm	C		0	0.0166667	0.09	0.1933333		0	0.0040825	0.0254951	0.0530723
		Lactate 0.6μm	D		0.01	0.03	0.11	0.2266667		0	0	0.0070711	0.0040825
		Lactate 0.6μm	E		0.02	0.0566667	0.1566667	0.3166667		0	0.0040825	0.0108012	0.0177951
		Lactate 0.6μm	F		0.01	0.0366667	0.11	0.2266667		0	0.0040825	0.0122474	0.0248328
		Lactate 0.6μm	G		0.0066667	0.0066667	0.13	0.2566667		0.0040825	0.008165	0.0141421	0.0362859
		Lactate 0.6μm	H		0.02	0.0366667	0.1733333	0.34		0.0070711	0.0177951	0.0285774	0.0561249
		Lactate 0.6μm	I		0.0066667	0.0066667	0.11	0.21		0.0040825	0.0108012	0.0212132	0.0463681
with Cell	Figure 5.	Lactate 0.6μm	J		0	-0.016667	0.07	0.1466667		0	0.0040825	0.0141421	0.0294392
					6 h	24 h	48 h	72 h		6 h	24 h	48 h	72 h
					mean	mean	mean	mean		S.E.	S.E.	S.E.	S.E.
		NH4+ 0.6μm	K		0.1466667	0.2266667	0.3933333	0.5133333		0.0040825	0.0177951	0.0470815	0.0163299
		NH4+ 0.6μm	L		0.1466667	0.2	0.3566667	0.4		0.0040825	0.0141421	0.0204124	0.0187083
		NH4+ 0.6μm	M		0.1433333	0.1966667	0.3066667	0.3666667		0.0040825	0.0147196	0.0285774	0.0216025
		NH4+ 0.6μm	N		0.1366667	0.1833333	0.32	0.3833333		0.008165	0.0147196	0.0070711	0.0227303
		NH4+ 0.6μm	O		0.1366667	0.2066667	0.3366667	0.4366667		0.0108012	0.0227303	0.0040825	0.0147196
		NH4+ 0.6μm	P		0.14	0.19	0.33	0.3866667		0	0.0070711	0.0187083	0.0147196
		NH4+ 0.6μm	Q		0.14	0.18	0.3333333	0.42		0.0470815	0.0141421	0.0147196	0.0254951
		NH4+ 0.6μm	R		0.1433333	0.1933333	0.3633333	0.4666667		0.0734847	0.0070711	0.0318852	0.0815475
		NH4+ 0.6μm	S		0.1266667	0.1666667	0.3233333	0.3833333		0.0441588	0.0122474	0.0108012	0.0530723
		NH4+ 0.6μm	T		0.1266667	0.1533333	0.2766667	0.3333333		0.0681909	0.0040825	0.0326599	0.0177951
						24 h	48 h	72 h			24 h	48 h	72 h
Medium	Figure 7.					mean	mean	mean			S.E.	S.E.	S.E.
		EV in VTCP 1.0μm				7%	16%	19%			0%	6%	3%
		EV in HTCP 1.0μm				23%	34%	41%			3%	2%	6%
				0 h	6 h	24 h	48 h	72 h		6 h	24 h	48 h	72 h
				mean	mean	mean	mean	mean		S.E.	S.E.	S.E.	S.E.
		NH4+ 0.6μm	A	200	108.49673	100	98.484848	100.72402		1.1320594	1.0188534	0	0.763018
		NH4+ 0.6μm	B	0	93.464052	98.235294	98.484848	100.8092		1.1320594	1.0188534	1.5151515	1.526036
		NH4+ 0.6μm	C	300	135.45455	100.76923	94.83871	97.086777		4.1659779	2.6646936	0	0.6855645
		NH4+ 0.6μm	D	0	99.090909	94.615385	93.548387	95.971074		4.1659779	2.3076923	1.1174521	0.9116057
		NH4+ 0.6μm	E	0	76.363636	90.769231	92.903226	95.929752		2.7272727	1.3323468	0	1.5789474
		NH4+ 0.6μm	F	0	92.727273	91.538462	90.967742	96.714876		0	1.3323468	1.9354839	0.9116057
Medium	Supplemental Figure A7	NH4+ 0.6μm	G	300	112.72727	93.076923	90.967742	96.301653		1.5745916	2.6646936	1.9354839	0.9116057
		NH4+ 0.6μm	H	0	93.636364	91.538462	92.258065	96.528926		1.5745916	1.3323468	2.9565004	0.9116057
				0 h	6 h	24 h	48 h	72 h		6 h	24 h	48 h	72 h
				mean	mean	mean	mean	mean		S.E.	S.E.	S.E.	S.E.
		NH4+ 0.03μm	I	200	149.6732	109.41176	104.54545	100.91567		1.1320594	4.6689729	1.5151515	2.6431718
		NH4+ 0.03μm	J	0	51.633987	80.588235	96.464646	99.63799		1.1320594	7.9574996	2.3144322	1.3215859
		NH4+ 0.03μm	K	300	205.45455	135.38462	123.87097	99.380165		1.5745916	6.6617339	12.087093	7.4618141
		NH4+ 0.03μm	L	0	68.181818	80	101.93548	97.004132		2.7272727	4.8038446	8.9396171	7.2928455
		NH4+ 0.03μm	M	0	31.818182	48.461538	84.516129	95.929752		1.5745916	20.117995	10.659814	8.696164

Table S3. Raw data of the results An Excel file is available online from the MDPI.
